# A Novel Nanobody-Horseradish Peroxidase Fusion Based-Competitive ELISA to Rapidly Detect Avian Corona-Virus-Infectious Bronchitis Virus Antibody in Chicken Serum

**DOI:** 10.3390/ijms23147589

**Published:** 2022-07-08

**Authors:** Kui Gu, Zengxu Song, Peng Ma, Ziwei Liao, Ming Yang, Changyu Zhou, Chao Li, Yu Zhao, Hao Li, Xin Yang, Changwei Lei, Hongning Wang

**Affiliations:** 1 Key Laboratory of Bio-Resource and Eco-Environment of Ministry of Education, College of Life Sciences, Sichuan University, Chengdu 610064, China; gukui0404@stu.scu.edu.cn (K.G.); szxtf2019@163.com (Z.S.); ma_peng99@163.com (P.M.); liaoziweivv@163.com (Z.L.); cemlia1127@163.com (M.Y.); ljl20131077@163.com (C.Z.); lichaomeet@gmail.com (C.L.); 2020322040035@stu.scu.edu.cn (Y.Z.); lihaoibv@163.com (H.L.); yangxin0822@163.com (X.Y.); 2 Animal Disease Prevention and Food Safety Key Laboratory of Sichuan Province, Chengdu 610064, China

**Keywords:** avian coronavirus, infectious bronchitis virus, nanobody, phage display technology, competitive ELISA

## Abstract

Avian coronavirus-infectious bronchitis virus (AvCoV-IBV) is the causative agent of infectious bronchitis (IB) that has brought great threat and economic losses to the global poultry industry. Rapid and accurate diagnostic methods are very necessary for effective disease monitoring. At the present study, we screened a novel nanobody against IBV-N protein for development of a rapid, simple, sensitive, and specific competitive ELISA for IBV antibody detection in order to enable the assessment of inoculation effect and early warning of disease infection. Using the phage display technology and bio-panning, we obtained 7 specific nanobodies fused with horseradish peroxidase (HRP) which were expressed in culture supernatant of HEK293T cells. Out of which, the nanobody of IBV-N-Nb66-vHRP has highly binding with IBV-N protein and was easily blocked by the IBV positive serums, which was finally employed as an immunoprobe for development of the competitive ELISA (cELISA). In the newly developed cELISA, we reduce the use of enzyme-conjugated secondary antibody, and the time of whole operation process is approximately 1 h. Moreover, the IBV positive serums diluted at 1:1000 can still be detected by the developed cELISA, and it has no cross reactivity with others chicken disease serums including Newcastle disease virus, Fowl adenovirus, Avian Influenza Virus, Infectious bursal disease virus and Hepatitis E virus. The cut-off value of the established cELISA was 36%, and the coefficient of variation of intra- and inter-assay were 0.55–1.65% and 2.58–6.03%, respectively. Compared with the commercial ELISA (IDEXX kit), the agreement rate of two methods was defined as 98% and the kappa value was 0.96, indicating the developed cELISA has high consistency with the commercial ELISA. Taken together, the novel cELISA for IBV antibody detection is a simple, rapid, sensitive, and specific immunoassay, which has the potential to rapidly test IBV antibody contributing to the surveillance and control of the disease.

## 1. Introduction

Infectious bronchitis (IB) is caused by the avian coronavirus-infectious bronchitis virus (AvCoV-IBV), which is recognized as a severe threat to the poultry industry globally owing to that it affects chickens of all ages and causes respiratory and nephrotic syndromes in broilers, as well as impairs egg production in layers and breeders [1,2,3]. AvCoV-IBV that belongs to the genus Gamma-coronavirus within the family of Coronaviridae in the order Nidovirales, is an enveloped, positive single-stranded RNA virus [4]. The 27 to 28 kb genome of AvCoV-IBV encodes several structural proteins, including spikes (S), membranes (M), envelopes (E), and nucleocapsids (N) protein [5,6]. Out of which, the S protein, a viral surface glycoprotein, elicited a neutralizing antibody response, while the N protein elicited a robust antibody response [6]. The diagnoses of IBV play an important role in the control of IB for the past few decades. Molecular diagnostic methods involving reverse transcription PCR or real-time PCR combined with sequencing are used to detect IBV in most laboratories, which benefits from its specificity and sensitivity, but requires expensive reagents and professional equipment [7]. Moreover, serological surveillance, which is used to measure viral exposure and vaccine efficacy in a flock, is critical for controlling the disease outbreak [8].

As a rapid, simple and sensitive method, conventional enzyme-linked immunosorbent assay (ELISA) that includes indirect ELISA, blocking ELISA, and competitive ELISA, has been widely used to detect the IBV antibody. To date, whole virus of IBV is used as the coated antigen for developing the ELISA that can detect antibodies against multiple viral structural proteins covering the S and N proteins. Nevertheless, whole IBV particles is tedious for producing procedures including virus proliferation and purification to cause the high price of the commercial ELISA kits, which limits its application [9]. Many studies indicated that recombinant S and N proteins from prokaryotic, yeast, or baculovirus systems could provide a rapid and large-scale detection method for IBV antibody infection [4]. The S protein of IBV has highly mutation in different IBV strains, which restricted its application in for detecting the IBV antibody. However, the N protein is the optimal candidate antigen for early diagnosis, both serological and molecular diagnosis, since it is highly conservative among the most IBV strains and can induce strong immune response for producing amount of antibody against N protein in early infection or immunization [4,8,10].

Recently, the nanobody produced from camelids or sharks, which constitutes the smallest antibody unit with perfect antigen-binding characteristics due to a molecular mass of roughly 15 kDa, continues to report for application in pathogens and antibody detection [11]. Compared to monoclonal antibodies, the nanobody is endowed with comparable affinity, abundant diversity, high thermal stability, easy production and the longer complementary determining region 3 (CDR3) for detecting the cryptic epitope of antigen [12]. Meanwhile, the development of nanobody-derivatives can also improve the detection performance of nanobody, such as nanobodies-fused reporter proteins (RANbodies) [13] and ferritin-fused nanobodies (Fenobodies) [14]. However, as of yet, there are no applications using the specific nanobodies to detect IBV antibody.

Based on the natural advantages of nanobodies, we screened the specific nanobodies against N protein of IBV (IBV-N) though five times immunization of Bactrian camels and the phage display technology in this study. Then, the specific nanobodies fused with horseradish peroxidase (HRP) against IBV-N protein were expressed in HEK293T cell lines. As a result, a direct competitive ELISA (cELISA) was developed for detecting the IBV antibody based on the recombinant IBV-N protein and the specific HRP-nanobodies. In this newly developed cELISA, the HRP-nanobodies against IBV-N protein was employed as an immunoprobe for directly compete the coated antigen with the IBV-seropositive sample, which can reduce the use of HRP-conjugate secondary antibody and shorten operation time. Furthermore, the established cELISA has high level of agreement for field samples detection compared with the commercial ELISA kit (IDEXX ELISA kit). It is a rapid, simple and sensitive cELISA to detect IBV antibodies for epidemiological investigation and antibody level monitoring.

## 2. Results

### 2.1. Expression, Purification and Identification of IBV-N Protein

**Figure 1:**
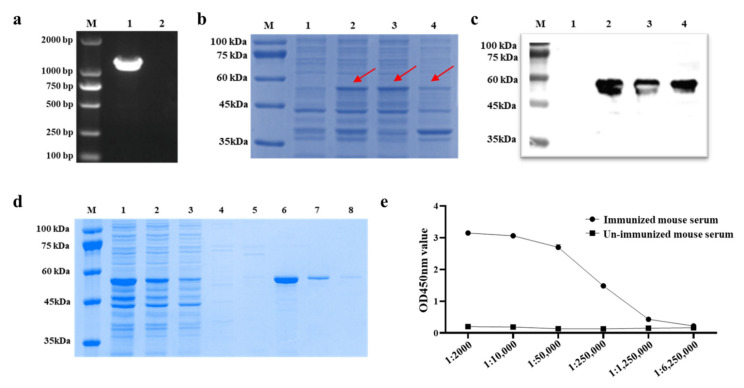
Expression, purification, and immunogenicity detection of IBV-N protein. (**a**) Amplification of the coding gene of IBV-N protein by PCR, around the target band of 1230 bp. (**b**) Expression and soluble analysis of IBV-N protein. M: Marker, Lane 1: pET-28a vector control, Lane 2: induced PET28a-IBV-N, Lane 3: the supernatant of induced PET28a-IBV-N, Lane 4: the inclusion body induced PET28a-IBV-N. Note: the red arrows point to protein expression of IBV-N protein. (**c**) Western blot analysis. (**d**) Purification of the IBV-N protein. Lane 1: the supernatant of un-purified IBV-N protein, Lane 2: the first liquid flowing from Ni-IDA column. Line 3–5: wash buffer for impurity, Line 6–8: elution buffer for target protein. (**e**) Analysis of the antigenicity of the IBV-N protein.

The coding gene of IBV-N protein (H120 strain) was amplified using PCR, and the target fragment band was about 1230 bp (Figure 1a). The results of SDS-PAGE showed that the recombinant IBV-N protein could be detected in supernatant of the induced *E. coli* strain after ultrasonic lysis (Figure 1b). Moreover, the target protein could be identified by Western blot using anti-His tag monoclonal antibody, with the expected size of 45 kDa (Figure 1c). Then, the highly purified IBV-N protein was obtained after purification (Figure 1d). After immunized three times, the titer of antibody against IBV-N protein in serum samples from the immunized mouse was detected by an indirect ELISA and it reached 1:1,250,000 (Figure 1d), suggesting that the IBV-N protein had a good antigenicity. These results indicated that we obtained the soluble IBV-N protein with high purity and strong antigenicity in this study.

### 2.2. Construction of a Phage Display VHH Library

**Figure 2:**
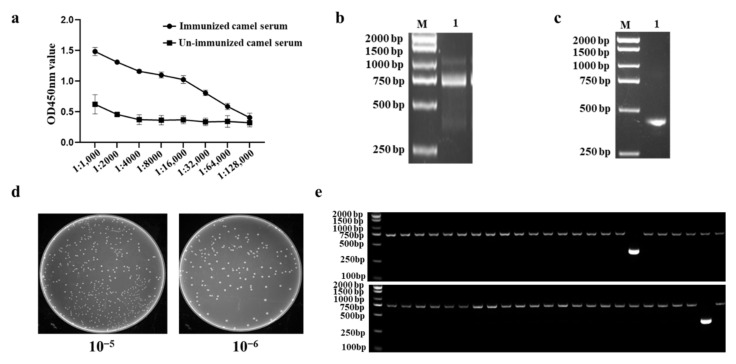
Construction of the VHH library against IBV-N protein. (**a**) Detection the antibody titer of serum samples from the immunized with IBV-N protein. (**b**) The first PCR fragment had a target band of about 700 bp; M: Marker DL2000, Lane 1: the first PCR products (~700 bp). (**c**) The VHH genes were re-amplified by second PCR, around 400 bp; M: Marker DL2000, Lane 1: the second PCR products (~400 bp). (**d**) The capacity of the VHH library was calculated by the plate count method, and it reached 8.0 × 10^9^ cfu/mL. (**e**) The correct insertion rate detected by PCR of 48 individual clones was nearly 96% (46/48).

After immunized five times with IBV-N protein, the titer of antibody in immunized camel serum reached 1: 64,000, showing that the immunized camel exhibited a good immune response against IBV-N protein (Figure 2a). The VHHs genes were amplified from peripheral blood mononuclear cells (PBMCs) of the immunized camel, and the target fragment around 700 bp was obtained by the first PCR (Figure 2b). Then, the products of the first PCR were employed as the template in second PCR to amplify VHH-only fragment, around 400 bp (Figure 2c). The capacity of VHH library was calculated by the plate count method, and the results showed the titer of the IBV-N-specific VHH library reached 8.0 × 10^9^ cfu/mL (Figure 2d). Meanwhile, 48 randomly selected individual colonies were performed by PCR and sequencing, and the results revealed that a correct insertion rate was 96% (46/48) (Figure 2e) and exhibited a high specificity and sequence diversity though sequencing (Data not shown). These results implied that a high quality immunized VHH library against IBV-N was successfully constructed in this study.

### 2.3. Screening and Sequencing of Nanobodies against IBV-N Protein

**Figure 3:**
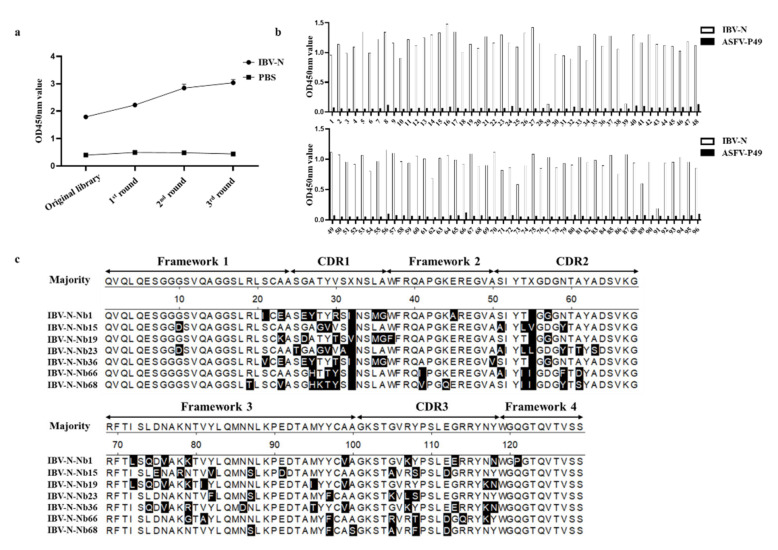
Screening the specific nanobodies against IBV-N protein by phage display technology. (**a**) Detection of specific phage enrichment with ELISA. (**b**) Identification of the periplasmic extracts from the 96 clones specifically binding to the IBV-N protein with indirect ELISA, and 93 clones were identified as positive (the P49 protein of African swine fever virus (ASFV-P49), which was expressed in *E. coli* (BL21) with the same expression method as IBV-N protein, was treated as the negative control). (**c**) Alignment of amino acid sequence of 7 screened nanobodies, and the sequences were grouped according to their CDRs (Shade residues with solid black that differ from the consensus).

After three rounds of bio-panning, the enrichment of phage particles was assessed by iELISA detection using an anti-M13 antibody and calculating the P/N values for each round of panning. The results suggested that the enrichment of phage continued to improve from the first round to the third round of screening (Figure 3a) and the ratio of positive/negative clones (P/N) increased from 9.8 to 2.4 × 10^3^ (Table 1). 93 clones from the randomly selected 96 clones were identified for expressing the specific nanobodies against IBV-N protein, and the positive rate reached 96.8% (93/96) (Figure 3b). After the 93 clones were sequenced, the 7 specific nanobodies against IBV-N protein were generated and termed IBV-N-Nb1, Nb15, Nb19, Nb23, Nb36, Nb66, Nb68, respectively (Figure 3c). These results indicated that we screened 7 specific nanobodies against IBV-N protein from the immunized VHH library.

### 2.4. Expression of RANbodies against IBV-N Protein

**Figure 4:**
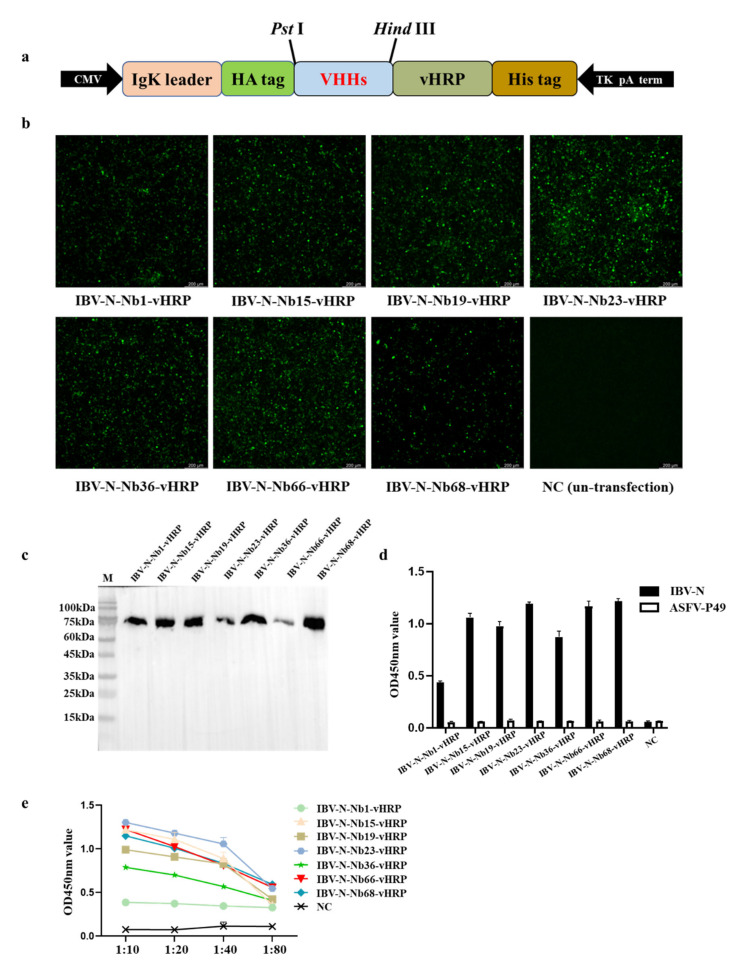
Expression and characterization of the 7 RANbodies against IBV-N protein in the HEK293T cells. (**a**) Schematic representation of the expression vector pCMV-VHHs-vHRP for RANbodies. (**b**) Identification of RANbodies against IBV-N protein expressed in the HEK293T cells by IFA (transfection for 48 h). (**c**) Western blot analysis of the 7 RANbodies against IBV-N protein secreted into the medium of HEK293T cells (transfection for 72 h). (**d**) Analysis of the 7 RANbodies specifically binding with the IBV-N protein with direct ELISA (transfection for 72 h), and the ASFV-P49 protein was treated as negative control. (**e**) Titers of the 7 RANbodies in the medium of HEK293T cells by direct ELISA detection. Note: the experiment of ELISA was repeated three times, and three repeat holes were performed for each experiment.

To produce the RANbodies against IBV-N protein, the 7 VHH genes were cloned into the pCMC-N1-vHRP vector, in which the recombinant plasmid included the IgK leader sequence, HA tag, the VHH coding gene, the codon optimized vHRP coding sequence and 6 × His tag (Figure 4a). After the recombinant plasmids were transfected for 48 h, the expression of 7 nanobodies were detected by IFA using an anti-HA mouse monoclonal antibody, suggesting that the 7 IBV-N-specific RANbodies could be expressed in HEK293T cells (Figure 4b). Furthermore, the secreted RANbodies in supernatant of medium were performed through Western blot and direct ELISA at the recombinant plasmids transfection for 72 h, and the results showed that the 7 IBV-N-specific RANbodies could be produced in medium (Figure 4c) and still reacted with IBV-N protein (Figure 4d). Meanwhile, the titer of RANbodies in medium were detected by direct ELISA. Out of which, 4 of RANbodies including IBV-N-Nb15-vHRP, IBV-N-Nb23-vHRP, IBV-N-Nb66-vHRP, IBV-N-Nb68-vHRP, exhibited a stable binding ability with IBV-N protein (Figure 4e). These results suggested we obtained 7 specific RANbodies secreted in medium against IBV-N protein.

### 2.5. Development the cELISA for Detecting IBV Antibody

**Figure 5:**
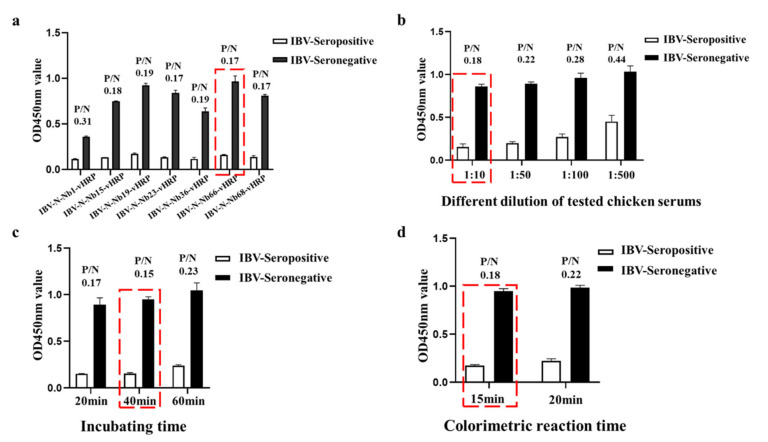
Optimization of the reacting conditions of the established cELISA. (**a**) Determination of the optimal blocking RANbody for developing the cELISA. (**b**) Different dilution of the tested chicken serums was detected for determination of the optimal dilution of the tested serum. (**c**) Determination of the incubation time of the cELISA. (**d**) The colorimetric reaction time was optimized. Note: (IBV-Seropositive samples (*n* = 6) and IBV-Seronegative samples (*n* = 6)).

Based on the conventional cELISA, the novel antibody detection on basis of the IBV-N-specific RANbodies cELISA was developed by advantage of the HRP-fused nanobodies, which could be directly applied to detect anti-IBV antibody and eliminated the use of HRP-conjugate secondary antibody. All the conditions were optimized with step by step. Firstly, the IBV-N-Nb66-vHRP was determined as the optimal competitive antibody with IBV-seropositive samples for development of the cELISA, in which the ratio of positive samples value/negative sample (0.17) was lower than other RANbodies for developing the cELISA (Figure 5a). Secondly, the results of a checkerboard titration assay suggested that the optimal amount of IBV-N protein was 800 ng/well, and the dilution of IBV-N-Nb66-vHRP protein was 1:16 (Table 2). Thirdly, the different dilutions of testing chicken serum in the cELISA with positive and negative sera showed that the best dilution of chicken serum was 1:10 (Figure 5b). Finally, the optimal incubation time for antigen-antibody reaction was determined as 40 min (Figure 5c), and the P/N value (0.15) was lowest in these three points. After adding TMB for colorimetric reaction, the P/N value (0.18) is the smallest after incubation time for 15 min (Figure 5d). All conditions were optimized and determined, the procedure of cELISA for IBV antibody detection was performed as followed: the 96-well ELISA plates were coated with IBV-N protein (800 ng/well) in 0.01 mol/L phosphate buffer (PBS) at 4 °C for overnight. After washing 3 times with PBST, the coated wells were blocked with 3% skimmed-milk at 37 °C for 1 h. Washed 3 times with PBST again, 100 µL testing mixtures containing 50 µL serum sample (diluted 1:10 with PBS) and 50 µL IBV-N-Nb66-vHRP protein (diluted 1:16 with PBS) were added to the wells and incubated 40 min at 37 °C. Following washed 3 times again, TMB (100 µL/well) was added and incubated in dark condition for 15 min at RT. Finally, the colorimetric reaction was terminated with 3 mol/L H_2_SO_4_ (50 µL/well), and the value of OD_450nm_ was read in less than ten minutes using a Multiskan FC microplate reader (Thermo Fisher Scientific, Waltham, MA, USA).

### 2.6. Cut-Off Values for the cELISA

**Figure 6:**
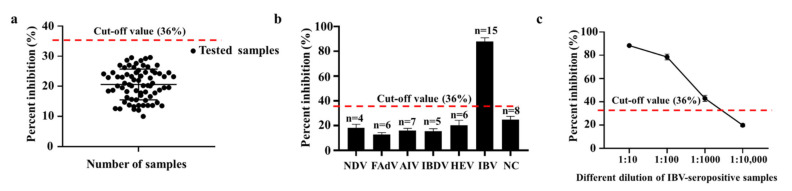
Specificity and sensitivity the cELISA for detecting anti-IBV antibodies. (**a**) 72 IBV-seronegative serum samples were tested using the cELISA for calculating the cut-off value. (**b**) Assessment of the cELISA detecting the antibodies against other chicken disease viruses, including NDV (*n* = 4), FAdV (*n* = 6), AIV (*n* = 7), IBDV (*n* = 5) and HEV (*n* = 6). (**c**) Determination of the largest dilution of positive chicken serum for anti-IBV antibodies by the developed cELISA.

To determine the cut-off value of the cELISA, 72 serum samples from the SPF chickens were performed using the developed cELISA. The results indicated that the average PI (%) of the negative serum samples was 21% with the standard deviation (SD) of the blocking rates determining as 5%, and the cut-off value for cELISA was 36% (21.0% + 3SD) (Figure 6a). In other words, when the PI value of the tested chicken serum was equal or greater than 36%, it was determined as the IBV-seropositive sample, vice versa.

### 2.7. Specificity, Sensitivity and Reproducibility of the cELISA

Positive serums against other chicken disease viruses including NDV, FAdV, AIV, IBDV and HEV were performed for evaluating the specificity of the cELISA. The results showed that only IBV-seropositive samples could compete the coated IBV-N protein with IBV-N-Nb66-vHRP protein, but other positive serums did not (Figure 6b). To determine the sensitivity of the developed cELISA, IBV-seropositive samples at different concentrations was detected using the cELISA, and the PI value was still greater than 36%, when the IBV-seropositive samples were diluted to 1:1000 (Figure 6c). For the most IBV-seropositive samples, the largest dilution was 1:1000 for detecting the anti-IBV antibodies. For the repeatability experiment, six samples selected randomly from the clinical chicken serum were detected by the cELISA for determining the intra- and inter-assay CV, which was 0.55–1.65% (mean value 1.10%) and 2.58–6.03% (mean value 4.17%), respectively (Table 3). These results demonstrated that the developed cELISA exhibited a good specificity, sensitivity and reproducibility for IBV antibody detection.

### 2.8. Agreements of the cELISA with a Commercial ELISA Kit

For evaluating whether the developed cELISA can be used to detect clinical samples, 350 clinical chicken serum samples that involved 150 serum samples with unknown exposure to the IBV virus or vaccine immunization and a total of 200 serum samples form chicken immunized with a live attenuated vaccine strain of H120 strain were used to compare the concordance rate between the developed cELISA and a commercial ELISA kit. For the 150 serum samples with unknown exposure to the IBV virus or vaccine immunization, 83 serum samples were the IBV-positive samples, and 67 serum samples were determined to be the IBV-negative samples using the developed cELISA. Nevertheless, 84 chicken serum samples were diagnosed as the IBV-seropositive samples and 66 samples were determined to IBV-seronegative samples through the commercial ELISA kit (Table 4). For the 200 serum samples form chicken immunized with a live attenuated vaccine strain of H120 strain, 195 chicken serum samples showed consistent results under the two methods (Table 4). As a result, the agreement rate of two methods was determined to be 98% (343/350) in these 350 clinical chicken serum samples (Table 4). Statistical analysis suggested that the tested results of the cELISA had a high level of consistency with commercial kits and the kappa value was 0.94 (Table 4). These results demonstrated that the established cELISA had a good application prospect in detecting IBV antibody.

## 3. Discussion

AvCoV-IBV is a serious respiratory pathogen, which causes enormous economic losses in the commercial poultry sector worldwide [15,16,17]. As a result, rapid and sensitive detection methods were critical for performing epidemiological investigation and immunization effectiveness assessment. In view of the above requirements, ELISA is a first choice in large-scale monitoring disease outbreaking. Nowadays, the different formats of ELISA are developed for IBV antibody monitoring. Out of which, an indirect ELISA based on whole virus particles or recombination N protein of IBV requires the HRP-polyclonal antibody against chicken IgY as the second antibody [18,19], leading to some troubling episodes such as virus cultivation and cross reactivity. In addition, other traditional ELISA based on monoclonal antibodies for developing a competitive ELISA or blocking ELISA, the performance requirements for monoclonal antibody are particularly high [20]. Whereas, the manufacturing processes of monoclonal antibody are complex and time-consuming, which exhibits low production and high costly. Nanobodies, a novel small molecule antibody, are the great alternatives to avoid the deficiency of traditional antibodies but can achieve the matching performance with monoclonal antibody [21]. In the recent years, nanobodies were widely applied to detect the specific targets including the bacteria [11,22,23], viruses [24,25,26], parasite [27], toxin [28], insecticide [29] and other target antigens [30,31,32,33,34,35,36].

**Figure 7:**
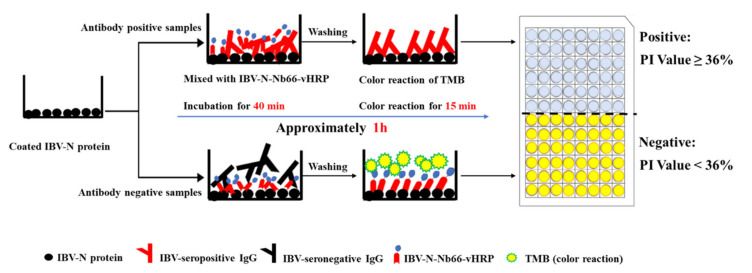
Schematic diagram of competitive ELISA test procedure for IBV antibody detection.

In the present study, the nanobodies are introduced for the first time to develop a cELISA for IBV antibody detection in chicken serum samples. Here, a phage display library against IBV-N protein was successfully generated though Bactrian camels’ immunization, and the specific nanobody of IBV-N-Nb66 against IBV-N protein was screened by bio-pinning of the three rounds, which was employed as a competitive immunoprobe for the development of the cELISA. Moreover, the coding gene of IBV-N-Nb66 was easily reconstructed in genetic platform and fused with the horseradish peroxidase for expression in HEK293T cell lines referring to the published reports [11,13], finally named IBV-N-Nb66-vHRP. Importantly, if the HEK293T cell lines expressing IBV-N-Nb66-vHRP were trained into stable cell lines, which will reduce costs and simplifies production further. For the newly developed cELISA, it can reduce the perform time and reagent costs due to the horseradish peroxidase directly fused with IBV-N-Nb66. This procedures of the established cELISA are shown in Figure 7, the time required of the cELISA is approximately 1 h, which is lower than other commercial ELISA assays (approximately 1.5 to 3 h). In addition, the developed cELISA shows high sensitivity, specificity, stability in IBV antibody monitoring.

The IDEXX kit that the whole virus antigens were coated in 96-well plates can be used to detect the antibody level in vaccination or other exposure to IBV, but it does not distinguish between animals vaccinated against IBV and those naturally exposed. In the present study, both the antibody of unknown exposure to the IBV virus and immunization of H20 strains were detected by the developed cELISA, the coincidence rate was 98% compared with the test results of the commercial ELISA. In summary, the newly established cELISA has great potential to be used for antibody titer evaluation of a live attenuated vaccine strain (H120 strain) in the future.

## 4. Materials and Methods

### 4.1. Serum Samples, Cells, and Vectors

72 serum samples from the specific pathogen free (SPF) chickens were used to determine the cut-off value of the developed cELISA in this study. 150 serum samples with unknown exposure to the IBV virus or vaccine immunization and a total of 200 serum samples form chicken immunized with a live attenuated vaccine strain of H120 strain were used to determine the coincidence rate between the cELISA and a commercial ELISA. In addition, positive serums of other avian viruses, including Newcastle disease virus (NDV), Fowl adenovirus (FAdV), Avian Influenza Virus (AIV), Infectious bursal disease virus (IBDV) and Hepatitis E virus (HEV) for evaluating the specificity of the cELISA were stored at our laboratory. HEK293T cells were cultured in Dulbecco’s modified Eagle’s medium (Life Technologies Corp, Carlsbad, CA, USA) supplemented with 10% fetal bovine serum (FBS, Gibco, Thermo Fisher Scientific, Waltham, MA, USA) and 100 IU/mL penicillin–streptomycin solution (Gibco USA). The PET-28a vector (Novagen, Sigma Aldrich, St. Louis, MO, USA) was employed for expression of the soluble IBV-N protein in the *E. coli* strain BL21(DE3). The pMECS vector and M13K07 help phage stored at our laboratory were used for VHH library construction. The pCMV-N1-vHRP vector was synthesized using the pEGFP-N1 vector as a backbone in a previous study [11], which was utilized to express the horseradish peroxidase-nanobodies in HEK293T cells in this study.

### 4.2. Experiment Animals

Six four-week-old Kunming mice were purchased from Chengdu Dossy Experimental Animals Co., LTD (Chengdu, China) for assessing the immunogenicity of N protein of IBV. A 4-year-old Bactrian camel was purchased from the Minqin Camel farm in Gansu province, China for preparation of the nanobodies library against IBV-N protein.

### 4.3. Expression, Purification and Identification of IBV-N Protein

The IBV-N protein was expressed in the *E. coli* strain BL21(DE3) according to the previous descriptions with some modifications [21]. In brief, the total RNA from a live attenuated vaccine strain (H120 strain) was extracted by TRIzol (Invitrogen, Carlsbad, CA, USA) following the manufacturer’s instructions and was reversely transcribed into cDNA using the PrimeScript™ RT reagent Kit (Takara, Beijing, China). A primer pair (Table S1) was designed according to the sequence of IBV-N protein coding gene (Genebank: FJ888351.1). Then, the coding gene of IBV-N protein amplified by PCR was digested with the restriction enzyme (*BamH* Ⅰ and *Xho* I) and ligated into PET-28a vector with T4 ligase (Takara, China). The positive recombinant plasmid named PET-28a-IBV-N was transformed into the *E. coli* strain BL21(DE3). Finally, the confirmed recombinant *E. coli* was induced with 0.2 mM isopropyl-β-dthiogalactoside (IPTG) for 10 h at 37 °C. After the collected bacteria were sonicated, the supernatant containing IBV-N protein was purified using the Ni-IDA 6FF Sefinose (TM) Resin Kit and Sephadex DeSalting Gravity Column (Sangon Biotech, Shanghai, China) for removing imidazole. The expression and purification of IBV-N protein were performed by SDS-PAGE and Western blot. To assess the immunogenicity of the recombinant IBV-N protein, three 4-week-old mice were injected by intraperitoneal immunization for three times at 0th, 14th and 28th days. For the first time, the injectant was the IBV-N protein (100 μg/mouse) and Freund’s complete adjuvant (Sigma Aldrich, St. Louis, MO, USA) mixture at a 1:1 volume. For the last two immunizations, the IBV-N protein (100 μg/mouse) was mixed with Freund’s incomplete adjuvant (Sigma Aldrich, St. Louis, MO, USA) at the same method above. After seven days of the last immunization, the titration of serum was performed by indirect ELISA (iELISA) and HRP-labeled goat anti-mouse antibody (PROTEINTECH GROUP, Wuhan, China) was treated as the secondary antibody.

### 4.4. Immunization of Camel

A healthy 4-year-old male Bactrian camel was immunized for five times based on previously reported procedures with some modifications [37,38]. Briefly, the healthy Bactrian camel was immunized with a single dose of 2 mL IBV-N protein (1 mg/mL), which was mixed with the same volume of Freund’s complete adjuvant for the first immunization and mixed the Freund’s incomplete adjuvant for the following four times. After the last immunization, the titration of the antibody against IBV-N protein in serum sample was determined by an iELISA, in which the IBV-N protein was treated as the coating antigen and the HRP-conjugated rabbit anti-camel IgG (SE283, Solarbio, Beijing, China) was used as a secondary antibody.

### 4.5. Generation of VHH Library

After the last immunization, the total of RNA from the peripheral blood mononuclear cells that were isolated from a 200 mL blood sample was extracted using TRIzol lysis method, and the cDNA was synthesized by reverse transcription. The variable regions of heavy-only chain antibodies were amplified by a two-steps PCR [37]. For the first round PCR, the fragment of about 700 bp was amplified with the primer pairs of CALL001 and CALL002 (Table S1). From these PCR products, the VHH genes were obtained through the second PCR with the primer pairs of VHH-FOR and VHH-REV (Table S1). The fragments of the second PCR around 400 bp were digested with the *Pst* I and *Not* I (NEB, Beijing, China) restriction enzymes and then ligated into the pMECS vector. Finally, the recombinant pMECS vectors named pMECS-VHHs were electroporated into TG1 competent cells. Then, the total of the electroporation TG1 cells were coated on the LB plates supplemented with 2% (*w*/*v*) final concentration of glucose and 100 µg/mL ampicillin. After overnight culture, all bacteria were collected in 15 mL centrifuge tube through a cell scraper. Here, a VHH library against IBV-N protein was successfully constructed and its capacity was evaluated with the plate count method. We randomly selected 48 colonies to detect the insertion rate of VHH gene by colony PCR using the primer pairs of MP57 and GIII (Table S1). Subsequently, the positive colonies were sequenced for identifying diversity. Finally, the VHH library against IBV-N protein was stored in LB medium supplemented with 20% glycerol and 100 µg/mL ampicillin at −80 °C until further use.

### 4.6. Screening of Specific VHH against IBV-N Protein

Phage rescue and three rounds of bio-panning were performed as described previously [37]. For the biopanning, the IBV-N protein (30 μg, 10 μg, or 5 μg) was used for the coated antigen from the first round to the third round of screening, respectively. After three rounds of bio-panning, the input and output phages in every round were quantified through the plate count method, and the IBV-N specific phage particles were evaluated by polyclonal phage ELISA using an anti-M13 antibody (Hangzhou HuaAn Biotechnology Co., Ltd., Hangzhou, China) as the second antibody.

Moreover, 96 colonies were randomly selected to analysis the expression of specific nanobodies that were separately cultured in TB medium with 100 µg/mL ampicillin and induced with 1 mmol/L isopropyl-β-d-thiogalactoside (IPTG). Then, periplasmic extracts of the induced bacteria were performed by an iELISA with mouse anti-HA monoclonal antibody (Sino Biological, Inc, Beijing, China). OD_450nm_ values ≥ 0.5 and the ratios of P (positive OD_450nm_)/N (negative OD_450nm_) greater than 3 were regarded as positive clones. All positive clones were sequenced, and the VHHs against IBV-N were grouped based on their complementary determining regions (CDRs) amino acid sequence.

### 4.7. Production of Nanobody-Horseradish Peroxidase Fusion against IBV-N Protein

Nanobodies-fused reporter protein (RANbodies) against IBV-N protein was expressed in HEK293T cells according to a previous description [11,13]. Briefly, the VHH genes against IBV-N protein were amplified from the positive clones containing pMECS-VHH vector by PCR using the primer pairs Nb-vHRP-F and Nb-vHRP-R (Table S1), and the production of PCR was ligated into the pCMV-N1-vHRP vector (Figure S1) by *Pst* I and *Hind* III digestion sites. Finally, the positive recombinant plasmids were subsequently transinfected into the HEK293T cells by Lipo8000™ Transfection Reagent (Beyotime Biotechnology, Shanghai, China). After 48 h of transfection, indirect immunofluorescence assay (IFA) was performed for determining whether the expression of RANbodies against IBV-N protein. After 72 h of transfection, the supernatants containing the specific RANbodies were detected with direct ELISA and western blot. Both IFA and western blot employed mouse anti-HA monoclonal antibody as the second antibody. Finally, the secreted RANbodies against IBV-N protein that contained the human Ig kappa chain signal peptide, HA tag, nanobodies coupled with the codon-optimized HRP, and the His tag were supplemented with 0.02% NaN_3_ (*w*/*v*) and stored at 4 °C for direct use.

### 4.8. Development of the Competitive ELISA using RANbodies

Based on the RANbodies against IBV-N protein, the competitive ELISA (cELISA) for detecting anti-IBV antibody was designed [39]. Firstly, the optimal RANbodies used to establish the cELISA was determined according to the following steps. The IBV-N protein (400 ng/well) was coated in the 96-well plate at 4 °C for overnight. After blocked with 3% skimmed-milk, the 50 μL of RANbodies mixed with 50 μL of IBV-Seropositive or IBV-Seronegative were incubated in the 96-well plate at 37 °C for 1 h to compete the IBV-N protein antigen. After washing three times with phosphate containing 0.5% *w/v* Tween-20 (PBST), 100 μL/well of TMB (Solarbio, Beijing, China) was used for a colorimetric reaction in the dark at 37 °C for 15 min. Finally, the positive value/negative value (P/N) of OD_450nm_ was the lowest, which this RANbody was determined as the optimal competitive antibody. Secondly, a checkerboard titration with direct ELISA was used to determine the optimal coated antigen and dilution of RANbody. Different amount of coated antigen including 200 ng, 400 ng, 800 ng and 1600 ng/well and the dilution ratios of RANbody (1:2, 1:4, 1:8, 1:16, 1:32, 1:64, 1:128, 1:256) were performed by a direct ELISA. The optimal condition was determined when the OD_450nm_ value was close to 1.0. Thirdly, the dilution of the tested porcine sera was also optimized. Six separate IBV-Seropositive and IBV-Seronegative were diluted 1:10, 1:50, 1:100, 1:500 for cELISA detection. Lastly, the competitive time of the test porcine serum and the RANbody with IBN-N protein antigen (20 min, 40 min and 60 min) and the colorimetric reaction of TMB (15 min and 20 min) were determined. When the smallest ratio of OD_450nm_ values between the positive and negative serum (P/N) was reached, the optimized conditions were obtained. All the conditions were optimized, the developed cELISA was determined.

### 4.9. Cut-off Value

72 serum samples from the SPF chickens were used to determine the cut-off value of the established cELISA in this study. The percent competitive inhibition (PI) was calculated according to the following formula: PI (%) = [1 − (OD_450nm_ value of testing serum sample/OD_450nm_ value of negative sample)] × 100% [40]. Furthermore, the cut off value of the cELISA was defined as the mean PI of 72 negative serum samples plus 3 standard deviations (SD), which could provide 95% or 99% confidence that the negative serum samples were within the set range.

### 4.10. Sensitivity, Specificity and Repeatability of cELISA

The specificity of the cELISA, serums positive for other common avian pathogens that included NDV, FAdV, AIV, IBDV and HEV were tested using the cELISA. The sensitivity of the developed cELISA was evaluated. Eight separate IBV-Seropositive and IBV-Seronegative detected by a commercial antibody kit (IDEXX) were diluted to different concentrations (1:10, 1:100, 1:1000, and 1:10,000) for the cELISA detection. To determine the repeatability of cELISA, six samples selected randomly from the clinical chicken serum were performed for evaluating the intra-assay (between plates) and inter-assay variabilities (within a plate), in which the results were presented as the coefficient of variation (CV), and the CV was the ratio of the SD to the mean OD_450nm_ value of each group of samples [41].

### 4.11. Comparisons of the cELISA with the Commercial ELISA Kit

Randomly collected 150 clinical chicken serum samples with unknown exposure to the IBV virus or vaccine immunization were tested using the developed cELISA and the commercial ELISA kit and each sample was tested three times. A total of 200 serum samples form chicken immunized with a live attenuated vaccine strain of H120 strain were also detected using the two methods. Then, the coincidence rates between cELISA and the commercial ELISA kit were calculated by the ratio of the number of the same decision of two methods to the number of total serum samples.

### 4.12. Statistical Analysis

At least three times, each experiment was independently replicated. GraphPad Prism version 7.0 (GraphPad Software, San Diego, CA, USA) was used to present the data, which included a one-way analysis of variance (one-way ANOVA) and Turkey: compare all pairings of columns. All presented data were shown as the mean ± SD, where * *p* < 0.05; ** *p* < 0.01; *** *p* < 0.001; Using SPSS software, the Kappa values were obtained to determine the coincidence between the developed cELISA and the commercial ELISA kit.

## 5. Conclusions

In summary, the specific nanobodies against IBV were firstly reported and screened. In addition, the newly developed cELISA based on IBV-N-Nb66-vHRP is a novel, rapid, specific, and low-cost immunoassay for IBV antibody monitoring, which can be used for evaluating the clinical serum samples with its highly specificity, sensitivity and no cross-reactivity with others chicken disease serums.

## Figures and Tables

**Table 1:** Enrichment of phage particles against IBV-N protein specific nanobodies during three rounds of panning.

Round of Screening	Input (Pfu/Well)	P Output (Pfu/Well)	N Output (Pfu/Well)	Recovery (P/Input)	P/N
**1st Round**	5.0 × 10^10^	3.0 × 10^4^	3.06 × 10^3^	6.0 × 10^−5^	9.8
**2nd Round**	5.0 × 10^10^	6.0 × 10^5^	2.7 × 10^4^	1.2 × 10^−5^	22.2
**3rd Round**	5.0 × 10^10^	3.6 × 10^6^	1.5 × 10^5^	1.8 × 10^−4^	2.4 × 10^3^

**Table 2:** Determination of the optimal coating amount of IBV-N protein and the optimal dilution of IBV-N-Nb66-vHRP fusion protein.

Coated IBV-N Protein	Different Dilution of the IBV-N-Nb66-vHRP							
	1:2	1:4	1:8	1:16	1:32	1:64	1:128	1:256
**1600 ng/well**	1.544 ± 0.020	1.330 ± 0.008	1.207 ± 0.070	1.157 ± 0.106	0.833 ± 0.103	0.678 ± 0.002	0.421 ± 0.020	0.287 ± 0.002
**800 ng/well**	1.513 ± 0.020	1.313 ± 0.014	1.136 ± 0.069	**1.086 ±** **0.053**	0.742 ± 0.027	0.653 ± 0.005	0.313 ± 0.115	0.291 ± 0.001
**400 ng/well**	0.952 ± 0.005	0.834 ± 0.001	0.831 ± 0.010	0.779 ± 0.020	0.674 ± 0.050	0.479 ± 0.012	0.308 ± 0.030	0.210 ± 0.020
**200 ng/well**	0.543 ± 0.143	0.437 ± 0.061	0.387 ± 0.018	0.366 ± 0.001	0.321 ± 0.005	0.223 ± 0.020	0.141 ± 0.010	0.130 ± 0.017

**Table 3:** The results of the repeatability test using the developed competitive ELISA.

Item	Range	Mid-Value
**Coefficient of variation in intra-plates**	0.55–1.65%	1.10%
**Coefficient of variation between inter-plates**	2.58–6.03%	4.17%

**Table 4:** Comparisons of the developed cELISA with IDEXX ELISA kit by detecting clinical chicken serum samples.

Samples	Number	The Developed cELISA	IDEXX ELISA Kit		Agreement (%)	Kappa Value
			+	-		
**Chicken serum samples with unknown exposure to the IBV virus or vaccine immunization**	**83**	**+**	**82**	1	98.0% (343/350)	0.94
	67	-	2	65		
**Chicken serum samples with a live attenuated vaccine strain of H120 strain**	195	+	191	4		
	5	-	0	5		

Note: “+” represents antibody positive (IBV); “-” represents antibody negative (IBV).

## Data Availability

Not applicable.

## Supplementary Materials

The following supporting information can be downloaded at: https://www.mdpi.com/article/10.3390/ijms23147589/s1.
