# Supplementary material for: A Novel Nanobody-Horseradish Peroxidase Fusion Based-Competitive ELISA to Rapidly Detect Avian Corona-Virus-Infectious Bronchitis Virus Antibody in Chicken Serum
Source: Int J Mol Sci. 2022 Jul 8;23(14):7589. doi: 10.3390/ijms23147589 (PMC9321063; doi:10.3390/ijms23147589)
Supplement: Supplementary file 1 [file ijms-23-07589-s001.zip › ijms-1791121-supplementary.pdf]

Supplementary Information

A novel nanobody-horseradish peroxidase fusion based-competitive ELISA to rapidly detect Avian coronavirus-infectious bronchitis virus antibody in chicken serum

Kui Gu[1,2†], Zengxu Song[1,2†], Peng Ma[1,2], Chao Li[1,2], Ming Yang[1,2], Changyu Zhou[1,2], Ziwei Liao[1,2], Yu Zhao[1,2], Hao Li[1,2], Xin Yang[1,2], Changwei Lei[1,2*], Hongning Wang[1,2*]

[1] Key Laboratory of Bio-Resource and Eco-Environment of Ministry of Education, College of Life Sciences, Sichuan University, Chengdu, Sichuan Province, People's 649 Republic of China.

[2] Animal Disease Prevention and Food Safety Key Laboratory of Sichuan Province, Chengdu, People's Republic of China.

*Correspondence: Changwei Lei leichangwei@scu.edu.cn;

Hongning Wang whongning@163.com

[†]Kui Gu, and Zengxu Song contributed equally to this work and considered as co- first authors

Table S1 Primers used for this work and the underlying sequences in the primers were restriction sites.

Primers	Sequences (5'-3')
CALL001	GTCCTGGCTGCTCTTCTACAAGG
CALL002	GGTACGTGCTGTTGAACTGTTCC
VHH-FOR (*Pst* I)	CAGGTGCAGCTGCAGGAGTCTGGGGGAGR
VHH-REV (*Not* I)	CTAGTGCGGCCGCTGAGGAGACGGTGACCTGGGT
MP57	TTATGCTTCCGGCTCGTATG
GIII	CCACAGACAGCCCTCATAG
Nb-vHRP-F (*Pst* I)	ATACTGCAGCAGGTGCAGCTGCAGGAG
Nb-vHRP-R (*Hind* III)	AGTAAGCTTTGAGGAGACGGTGACCTG

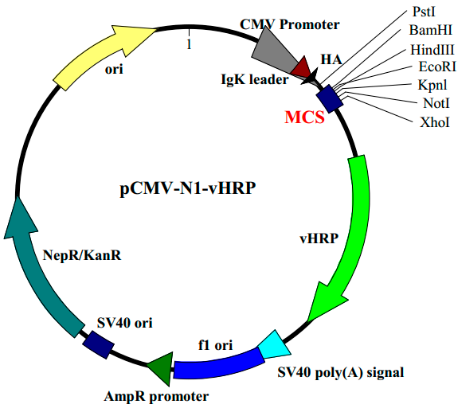

Figure S1 Plasmid profile of pCMV-N1-vHRP vector and the sequence was listed as following:

tagttattaatagtaatcaattacggggtcattagttcatagcccatatatggagttccgcgttacataacttacggtaaatggcc
cgcctggctgaccgcccaacgacccccgcccattgacgtcaataatgacgtatgttcccatagtaacgccaatagggacttt
ccattgacgtcaatgggtggagtatttacggtaaactgcccacttggcagtacatcaagtgtatcatatgccaagtacgcccc
ctattgacgtcaatgacggtaaatggcccgcctggcattatgcccagtacatgaccttatgggactttcctacttggcagtaca
tctacgtattagtcatcgctattaccatggtgatgcggttttggcagtacatcaatgggcgtggatagcggtttgactcacggg
gatttccaagtctccaccccattgacgtcaatgggagtttgttttggcaccaaaatcaacgggactttccaaaatgtcgtaaca
actccgccccattgacgcaaatgggcggtaggcgtgtacggtgggaggtctatataagcagagctggtttagtgaaccgtc
agatccgctagcatggagaccgacaccttactgctgtgggtgctgctgttatgggtgcccggtagcaccggcgactaccca

tacgatgttccagattacgctctgcagggatccaagcttgaattcggtaccctcgaggcggccgccagtagtagtggcagt

ggtccaccggtcgccaccatggtgagcaagggcgaggagctgttcaccggggtggtgcccatcctggtcgagctggac

ggcgacgtaaacggccacaagttcagcgtgtccggcgagggcgagggcgatgccacctacggcaagctgaccctgaa

gttcatctgcaccaccggcaagctgcccgtgccctggcccaccctcgtgaccaccctgacctacggcgtgcagtgcttcag

ccgctaccccgaccacatgaagcagcacgacttcttcaagtccgccatgcccgaaggctacgtccaggagcgcaccatct

tcttcaaggacgacggcaactacaagacccgcgccgaggtgaagttcgagggcgacaccctggtgaaccgcatcgagct

gaagggcatcgacttcaaggaggacggcaacatcctggggcacaagctggagtacaactacaacagccacaacgtctat

atcatggccgacaagcagaagaacggcatcaaggtgaacttcaagatccgccacaacatcgaggacggcagcgtgcag

ctcgccgaccactaccagcagaacacccccatcggcgacggccccgtgctgctgcccgacaaccactacctgagcacc

cagtccgccctgagcaaagaccccaacgagaagcgcgatcacatggtcctgctggagttcgtgaccgccgccgggatca

ctctcggcatggacgagctgtacaagtaaagcggccgcgactctagatcataatcagccataccacatttgtagaggttttac

ttgctttaaaaaacctcccacacctccccctgaacctgaaacataaaatgaatgcaattgttgttgttaacttgtttattgcagctt

ataatggttacaaataaagcaatagcatcacaaatttcacaaataaagcatttttttcactgcattctagttgtggtttgtccaaac

tcatcaatgtatcttaaggcgtaaattgtaagcgttaatattttgttaaaattcgcgttaaatttttgttaaatcagctcattttttaacc

aataggccgaaatcggcaaaatcccttataaatcaaaagaatagaccgagatagggttgagtgttgttccagtttggaacaa

gagtccactattaaagaacgtggactccaacgtcaaagggcgaaaaaccgtctatcagggcgatggcccactacgtgaac

catcaccctaatcaagttttttggggtcgaggtgccgtaaagcactaaatcggaaccctaaagggagcccccgatttagagc

ttgacggggaaagccggcgaacgtggcgagaaaggaagggaagaaagcgaaaggagcgggcgctagggcgctggc

aagtgtagcggtcacgctgcgcgtaaccaccacacccgccgcgcttaatgcgccgctacagggcgcgtcaggtggcact

tttcggggaaatgtgcgcggaacccctatttgtttatttttctaaatacattcaaatatgtatccgctcatgagacaataaccctg

ataaatgcttcaataatattgaaaaaggaagagtcctgaggcggaaagaaccagctgtggaatgtgtgtcagttagggtgtg

gaaagtccccaggctccccagcaggcagaagtatgcaaagcatgcatctcaattagtcagcaaccaggtgtggaaagtcc

ccaggctccccagcaggcagaagtatgcaaagcatgcatctcaattagtcagcaaccatagtcccgcccctaactccgcc

catcccgcccctaactccgcccagttccgcccattctccgccccatggctgactaattttttttatttatgcagaggccgaggc

cgcctcggcctctgagctattccagaagtagtgaggaggcttttttggaggcctaggcttttgcaaagatcgatcaagagac

aggatgaggatcgtttcgcatgattgaacaagatggattgcacgcaggttctccggccgcttgggtggagaggctattcgg

ctatgactgggcacaacagacaatcggctgctctgatgccgccgtgttccggctgtcagcgcaggggcgcccggttcttttt

gtcaagaccgacctgtccggtgccctgaatgaactgcaagacgaggcagcgcggctatcgtggctggccacgacgggc

gttccttgcgcagctgtgctcgacgttgtcactgaagcgggaagggactggctgctattgggcgaagtgccggggcagga

tctcctgtcatctcaccttgctcctgccgagaaagtatccatcatggctgatgcaatgcggcggctgcatacgcttgatccgg

ctacctgcccattcgaccaccaagcgaaacatcgcatcgagcgagcacgtactcggatggaagccggtcttgtcgatcag
gatgatctggacgaagagcatcaggggctcgcgccagccgaactgttcgccaggctcaaggcgagcatgcccgacggc
gaggatctcgtcgtgacccatggcgatgcctgcttgccgaatatcatggtggaaaatggccgcttttctggattcatcgactg
tggccggctgggtgtggcggaccgctatcaggacatagcgttggctacccgtgatattgctgaagagcttggcggcgaat
gggctgaccgcttcctcgtgctttacggtatcgccgctcccgattcgcagcgcatcgccttctatcgccttcttgacgagttctt
ctgagcgggactctggggttcgaaatgaccgaccaagcgacgcccaacctgccatcacgagatttcgattccaccgccgc
cttctatgaaaggttgggcttcggaatcgttttccgggacgccggctggatgatcctccagcgcggggatctcatgctggag
ttcttcgcccaccctagggggaggctaactgaaacacggaaggagacaataccggaaggaacccgcgctatgacggca
ataaaaagacagaataaaacgcacggtgttgggtcgtttgttcataaacgcggggttcggtcccagggctggcactctgtc
gataccccaccgagaccccattggggccaatacgcccgcgtttcttccttttccccaccccaccccccaagttcgggtgaa
ggcccagggctcgcagccaacgtcggggcggcaggccctgccatagcctcaggttactcatatatactttagattgatttaa
aacttcatttttaatttaaaaggatctaggtgaagatcctttttgataatctcatgaccaaaatcccttaacgtgagttttcgttcca
ctgagcgtcagaccccgtagaaaagatcaaaggatcttcttgagatcctttttttctgcgcgtaatctgctgcttgcaaacaaa
aaaaccaccgctaccagcggtggtttgtttgccggatcaagagctaccaactctttttccgaaggtaactggcttcagcaga
gcgcagataccaaatactgtccttctagtgtagccgtagttaggccaccacttcaagaactctgtagcaccgcctacatacct
cgctctgctaatcctgttaccagtggctgctgccagtggcgataagtcgtgtcttaccgggttggactcaagacgatagttac
cggataaggcgcagcggtcgggctgaacggggggttcgtgcacacagcccagcttggagcgaacgacctacaccgaa
ctgagatacctacagcgtgagctatgagaaagcgccacgcttcccgaagggagaaaggcggacaggtatccggtaagc
ggcagggtcggaacaggagagcgcacgagggagcttccagggggaaacgcctggtatctttatagtcctgtcgggtttcg
ccacctctgacttgagcgtcgatttttgtgatgctcgtcaggggggcggagcctatggaaaaacgccagcaacgcggcctt
tttacggttcctggccttttgctggccttttgctcacatgttctttcctgcgttatcccctgattctgtggataaccgtattaccgcc
atgcat
